# The Pro-Survival Oct4/Stat1/Mcl-1 Axis Is Associated with Poor Prognosis in Lung Adenocarcinoma Patients

**DOI:** 10.3390/cells10102642

**Published:** 2021-10-03

**Authors:** Yu-Chu Su, Yi-Cheng Chen, Yau-Lin Tseng, Gia-Shing Shieh, Pensee Wu, Ai-Li Shiau, Chao-Liang Wu

**Affiliations:** 1 Department of Otolaryngology, National Cheng Kung University Hospital, College of Medicine, National Cheng Kung University, Tainan 70403, Taiwan; yc05018@gmail.com; 2 Department of Biochemistry and Molecular Biology, College of Medicine, National Cheng Kung University, Tainan 70101, Taiwan; s58971081@gs.ncku.edu.tw; 3 Department of Medical Research, Ditmanson Medical Foundation Chiayi Christian Hospital, Chiayi 60002, Taiwan; 4 Division of Thoracic Surgery, Department of Surgery, College of Medicine, National Cheng Kung University, Tainan 70101, Taiwan; tsengyl@mail.ncku.edu.tw; 5 Department of Urology, Tainan Hospital, Ministry of Health and Welfare, Executive Yuan, Tainan 70043, Taiwan; s98881080@yahoo.com.tw; 6 Keele Cardiovascular Research Group, School of Medicine, Keele University, Staffordshire ST5 5BG, UK; p.wu@keele.ac.uk; 7 Academic Unit of Obstetrics and Gynaecology, University Hospital of North Midlands, Stoke-on-Trent, Staffordshire ST4 6QG, UK; 8 Department of Microbiology and Immunology, College of Medicine, National Cheng Kung University, Tainan 70101, Taiwan

**Keywords:** lung adenocarcinoma, anti-apoptosis, cell survival, Oct4, Stat1, Mcl-1

## Abstract

The embryonic stem cell marker Oct4 is expressed in several human cancers and is positively correlated with a poor outcome in cancer patients. However, its physiological role in cancer progression remains poorly understood. Tumor cells block apoptosis to escape cell death so that they can proliferate indefinitely, leading to ineffective therapy for cancer patients. In this study, we investigated whether Oct4 regulates the apoptosis pathway and contributes to poor prognosis in patients with lung adenocarcinoma. Our results revealed that Oct4 expression is correlated with Stat1 expression in lung adenocarcinoma patients and Oct4 is directly bound to the *Stat1* promoter to transactivate Stat1 in lung adenocarcinoma cells. Expression of the Stat1 downstream gene Mcl-1 increased in Oct4-overexpressing cancer cells, while Stat1 knockdown in Oct4-overexpressing cancer cells sensitized them to cisplatin-induced apoptosis. Furthermore, Oct4 promoted Stat1 expression and tumor growth, whereas silencing of Stat1 reduced Oct4-induced tumor growth in human lung tumor xenograft models. Taken together, we demonstrate that Oct4 is a pro-survival factor by inducing Stat1 expression and that the Oct4/Stat1/Mcl-1 axis may be a potential therapeutic target for lung adenocarcinoma.

## 1. Introduction

Lung cancer is the leading cause of death due to cancer in the world. Most patients with lung cancer have lung adenocarcinoma and their 5-year survival rate is approximately 10% [1]. Surgery is the general treatment for patients with early-stage lung adenocarcinoma, while late-stage lung adenocarcinoma patients require multidisciplinary treatment strategies, such as chemotherapy followed by surgery, or concurrent chemoradiotherapy [2]. Because of the difficulties in early detection and the high risk of local recurrence and distant metastasis, the prognosis of lung cancer is poor. Therefore, the development of effective treatment modalities for lung cancer is a matter of urgency.

Oct4, a transcription factor of the POU homeobox gene family, plays a key role in the self-renewal and pluripotency of embryonic stem cells [3,4,5]. In addition to stem cells, Oct4 is expressed in cancer cells, for example, in lung, breast, pancreas and bladder cancers [6,7,8,9]. Oct4 expression is correlated with tumor progression and poor patient prognosis [7,10]. We previously demonstrated that Oct4 promotes tumor metastasis through the Egr1/OPN axis in lung cancer [11]. Moreover, Oct4 is involved in modulating the tumor microenvironment. Oct4 promotes M2 polarization by increasing M-CSF secretion, thus stimulating tumor metastasis [12]. These data imply the critical role of Oct4 in tumor metastasis. During cancer progression, tumor cells block apoptosis to escape cell death so that they can proliferate indefinitely, leading to ineffective therapy of cancer patients. In this study, we investigated whether Oct4 regulates the apoptosis pathway and contributes to poor prognosis in patients with lung adenocarcinoma.

The transcription factor Stat1 functions as a regulator of cell proliferation, differentiation, inflammation and apoptosis [13]. When cytokines bind to the receptor, cytoplasmic Stat1 is phosphorylated and forms a homodimer or a heterodimer, which then translocates to the nucleus to initiate transcription. Traditional Stat1 signaling has pro-apoptotic functions [14]. However, recent works have shown that Stat1 expression is dysregulated in cancers, including lung cancer, melanoma and Wilms’ tumor [15,16,17]. Reduced migration and metastasis are detected in Stat1-knockdown melanoma cells [15]. Moreover, Stat1 overexpression is associated with the protection of cancer cells from radiation and chemotherapy [18,19]. Therefore, Stat1 may participate in some unidentified mechanisms to regulate tumor progression.

Apoptosis is the process of programmed cell death to maintain tissue homeostasis [20]. In malignant transformation, anti-apoptotic mechanisms cause uncontrolled cell proliferation [21]. Blocking the apoptotic pathway results in ineffective therapy and poor prognosis in cancer patients [22,23]. Given that apoptosis causes the least immune reaction [24], it is critical to better understand the anti-apoptotic mechanism in order to develop effective strategies for cancer treatment. Previous studies have revealed that both Oct4 and Stat1 are overexpressed in lung adenocarcinoma and might play a role in cell survival [7,12,16,17]. Computational analysis shows that Oct4 may bind to the *Stat1* promoter to transactivate Stat1 gene expression. Therefore, we hypothesized that Oct4 regulates the apoptotic pathway by upregulating Stat1 expression. In this study, we show that Stat1 expression is correlated with Oct4 expression in lung adenocarcinoma patients. We found that Oct4 enhances Stat1 expression by directly binding to the *Stat1* promoter. Moreover, Oct4 promotes tumor growth, whereas silencing of Stat1 expression reduces tumor growth in vivo. Our results support the critical role of the Oct4/Stat1/Mcl-1 axis in cell survival in lung adenocarcinoma.

## 2. Materials and Methods

### 2.1. Oncomine Database Analysis

The Oncomine cancer microarray database (https://www.oncomine.org, accessed on 9 March 2016) was used to compare the gene expression level of lung adenocarcinoma with normal tissues. We used the following filters: (1) cancer type, lung cancer; (2) analysis type, cancer vs. normal analysis. Among 12 gene expression profiles, Okayama Lung contains most lung adenocarcinoma cases. Expression of Oct4 and Stat1 were analyzed in 226 lung adenocarcinoma and 20 normal lung tissues.

### 2.2. Cell Lines

Human lung adenocarcinoma cell lines (A549 and H1299) were purchased from the Bioresource Collection and Research Center (Food Industry Research and Development Institute, Hsinchu, Taiwan). The short tandem repeat profiling of the cell lines was performed at the Center for Genetic Medicine of the NCKU and the use of the authenticated cell lines was verified. Cells were cultured at 37 °C in a 5% CO_2_ atmosphere in Dulbecco’s modified Eagle minimum essential medium (DMEM) supplemented with 10% cosmic calf serum (Hyclone, Logan, UT, USA), 2 mmol/L of l-glutamine and 50 μg/mL of gentamicin.

### 2.3. Plasmids

The lentiviral vector pSin-EF2-Oct4-Pur was purchased from Addgene (Cambridge, MA, USA) and the coding region of Oct4 was removed to generate the control vector pSin-EF2-Pur. The *Stat1* promoter region from −585 to +1545 bp relative to the transcription start site of Stat1 was amplified by PCR with the forward primer 5′-CGTTTAGGAGAAGCCCAGGTAAAGAAGCTG-3′ and the reverse primer 5′-AAAGAATTCAACCCAGTCACCAAATCATTTACTGTT-3′. The PCR product was digested with *Kpn*I and *Sal*I and inserted into a luciferase reporter vector pFRL2 [25], resulting in the plasmid pFRL2-Stat1p. Next, pFRL2-Stat1p was digested with *Kpn*I/*Mlu*I, *Kpn*I/*Xho*I and *Kpn*I/*Ale*I; the resulting large fragment was treated with T4 DNA polymerase and self-ligated to generate deletions of the *Stat1* promoter from +364 to +1545, from +598 to +1545 and from +1064 to +1545 bp, respectively. pFRL2-Stat1p, with a point mutation (T577G) in the Oct4-binding site, was generated by site-directed mutagenesis with the forward primer 5′-CGTTTTCTTCTTTTCGCAGAAAGTGTCATTTGC-3′ and the reverse primer 5′-CGAGGATGGCATACAGCAAATGACACTTTCT-3′. The DNA sequences of mutated vectors were confirmed by sequence analyses.

For knockdown experiments, pLKO.1-puro-based lentiviral vectors expressing short hairpin RNA (shRNA) specific for human Stat1 (TRCN0000004265 and TRCN0000004267) and luciferase (Luc) (TRCN0000072246) were obtained from the National RNAi Core Facility, Academia Sinica, Taiwan. Lentiviruses were produced as previously described [26] and viral titers expressed as lentiviral particles (LPs) were determined using the virus-associated p24 ELISA kit (QuickTiter Lentivirus titer kit; Cell Biolabs, San Diego, CA, USA).

### 2.4. Luciferase Reporter Assay

A549 and H1299 cells were cotransfected with pSin-EF2-Oct4-Pur and pFRL2-Stat1p using Lipofectamine 2000 (Invitrogen, Carlsbad, CA, USA) for 48 h. Cell lysates were collected to examine luciferase activity using the dual-luciferase reporter assay system (Promega Corporation, Madison, WI, USA). The relative luciferase activity was measured as firefly luciferase activity divided by *renilla* luciferase activity in order to normalize transfection efficiency.

### 2.5. Quantification of mRNA Expression

Total RNA was isolated from cells using the TRIzol reagent (Invitrogen, Carlsbad, CA, USA) and 1 μg of RNA was used for cDNA synthesis using a reversed iT first-strand synthesis kit (Thermo Fisher Scientific, Bremen, Germany) according to the manufacturer’s instructions. Quantitative real-time RT-PCR was performed using a Rotor-Gene Q system (Qiagen, Hilden, Germany). The following primers were used for RT-PCR: Oct4, 5′-GTCCGAGTGTGGTTCTGTA-3′ (forward) and 5′-CTCAGTTTGAATGCATGGGA-3′ (reverse); Stat1, 5′-TGTCTCGGATAGTGGGCTCTG-3′ (forward) and 5′-GCTGGCCTTTCTTTCATTTCC-3′ (reverse); GAPDH, 5′-ACTTCAACAGCGACACCCACT-3′ (forward) and 5′- GCCAAATTCGTTGTCATACCAG-3′ (reverse). The relative quantification of Oct4 and Stat1 mRNA was analyzed using the comparative CT method and normalized to that of GAPDH.

### 2.6. Immunological Assays

For immunoblotting, cell lysates were separated on 8–15% SDS-PAGE and transferred onto PVDF membranes (Merck Millipore, Burlington, MA, USA). The PVDF membranes were blocked with 5% BSA and then incubated with primary antibodies. After washing with PBST, the membranes were incubated with HRP-conjugated secondary antibodies (Jackson Immunoresearch, West Grove, PA, USA). The specific signal was detected using ECL reagents (Merck Millipore) and a UVP imaging system (Upland, CA, USA). For immunohistochemical staining, tumor tissues were fixed in 10% buffered formalin and paraffin-embedded sections were prepared. The sections were de-paraffinized in xylene, then dehydrated in ethanol and finally rehydrated in distilled water. After antigen retrieval, the sections were blocked with 5% BSA and then incubated with primary antibodies overnight. Then, HRP-conjugated secondary antibodies (Jackson Immunoresearch) and AEC reagent (Santa Cruz Biotechnology, Inc., Santa Cruz, CA, USA) were used to detect specific signals. The primary antibodies used for immunoblotting were Oct4 (Cell Signaling Technology, Beverly MA, USA), Stat1 (Santa Cruz), phospho-Stat1 (Tyr701) (Cell Signaling Technology), Mcl-1 (Santa Cruz Biotechnology, Inc.), cleaved caspase-3 (Cell Signaling Technology) and β-actin (Sigma-Aldrich, St. Louis, MO, USA).

### 2.7. Chromatin Immunoprecipitation (ChIP) Assay

ChIP was performed using the EZ-ChIP kit (Millipore, Billerica, MA, USA) according to the manufacturer’s instructions [11]. The following primers were used for PCR: with the Oct4 response element (ORE), 5′-GCCAGTCGTGCTCTGGCAGT-3′ (forward) and 5′-CGCACAGCACGTTAGGTGCCA-3′ (reverse); without the ORE, 5′-GCAGAGGTGTGGTTGATTGTGCT-3′ (forward) and 5′-TGTGAGTCCAGCATCCTCATTAAGC-3′ (reverse).

### 2.8. TUNEL Assay

A549 cells were fixed in 4% paraformaldehyde for 30 min. TUNEL staining was performed using the DeadEnd fluorometric TUNEL system (Promega Corporation). The percentage of apoptotic cells was calculated by dividing the number of TUNEL-positive cells by the number of DAPI-stained cells.

### 2.9. Animal Experiments

All procedures in the experiments adhered to the guidelines approved by the Laboratory Animal Care and Use Committee of National Cheng Kung University (NCKU).

Male NOD/SCID mice at 8 weeks of age were subcutaneously inoculated with A549-vector or A549-Oct4 cells (1 × 10^6^ cells). For knockdown and drug treatment experiments, tumor-bearing mice were intratumorally treated with 1 × 10^9^ LPs of LV-shLuc or LV-shStat1 at day 21 or fludarabine (2.5 μg) at day 14. Tumor volumes were measured every 3 days and calculated as length × width^2^ × 0.45 [27].

### 2.10. Statistical Analyses

The survival analysis in the human study was performed by Kaplan–Meier analysis and the log-rank test. Correlations were measured using Pearson correlation coefficient (r). Statistical significance between groups was assessed using a Student’s *t*-test or one-way analysis of variance (ANOVA). Differences in animal tumor growth were compared by two-way ANOVA with repeated measures.

## 3. Results

### 3.1. Oct4 and Stat1 Expression Is Elevated and Associated with Poor Prognosis of Human Lung Adenocarcinoma

**Figure 1:**
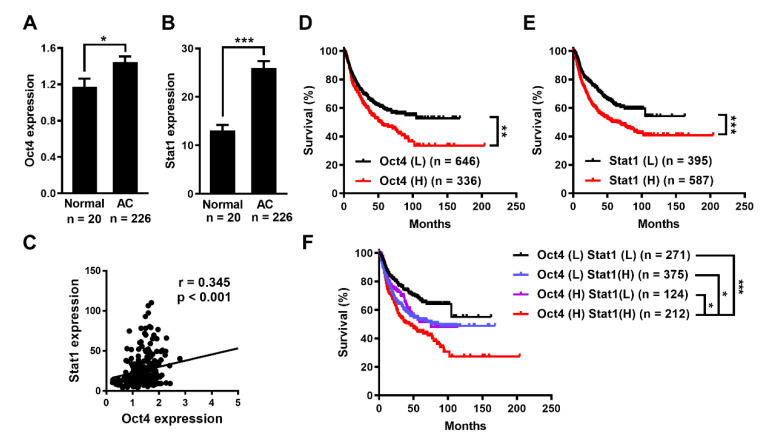
Oct4 is highly expressed in human lung adenocarcinoma and is correlated with Stat1 expression. (**A**,**B**) Comparison of Oct4 (**A**) and Stat1 (**B**) expression in a normal human lung and lung adenocarcinoma (AC). (**C**) Correlation of Oct4 and Stat1 was analyzed by Pearson correlation coefficient in human lung adenocarcinoma. Data were obtained from Oncomine.org (accession no. GSE31210). Data are the mean ± S.E.M. (**D**,**E**) Kaplan–Meier analysis of relapse-free survival in lung cancer patients according to the expression levels of Oct4 (**D**) and Stat1 (**E**). H, high expression; L, low expression. (**F**) Kaplan–Meier analysis of relapse-free survival in lung cancer patients with high (H) or low (L) expression levels of both Oct4 and Stat1. Data were obtained from the Kaplan–Meier plotter database (Affymetrix ID: 208286_x_at (Oct4) and M97935_MB_at (Stat1); cut-off value, 190 (Oct4) and 360 (Stat1)). Statistical differences were analyzed by the log-rank test. * *p* < 0.05, ** *p* < 0.01 and *** *p* < 0.001.

To compare Oct4 and Stat1 expression in lung adenocarcinoma patients with that in the controls, we analyzed the RNA expression in human lung adenocarcinoma in the Oncomine database (accession no. GSE31210) [28]. RNA levels of both Oct4 and Stat1 increased 1.2- and 1.6-fold, respectively, in the lungs of adenocarcinoma patients compared with the controls (Figure 1A,B). There was a positive correlation between Oct4 and Stat1 expression in the Oncomine data (Figure 1C). To further assess whether Oct4 or Stat1 expression is associated with the survival of patients with lung cancer, we analyzed the prognosis of lung cancer patients using the Kaplan–Meier plotter database (http://kmplot.com/analysis/, accessed on 1 June 2017) [29]. Patients with low expression of either Oct4 or Stat1 had longer relapse-free survival than those with high expression (Figure 1D,E). Moreover, patients with high expression of both Oct4 and Stat1 had poorer prognosis than those with low expression of both (Figure 1F). Taken together, these results show that Oct4 is associated with Stat1 expression and may be useful as a prognostic marker for lung adenocarcinoma.

### 3.2. Oct4 Transactivates the Stat1 Promoter

**Figure 2:**
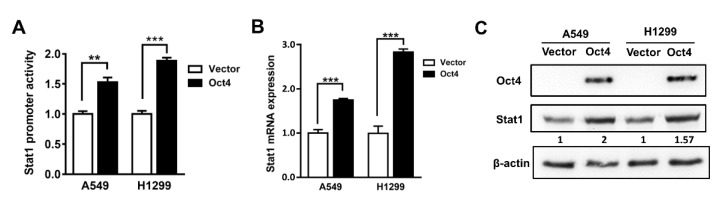
Oct4 induces Stat1 expression in lung adenocarcinoma cells. (**A**) A549 and H1299 cells were cotransfected with pFRL2-Stat1p and pSin-EF2-Oct4-Pur and then cultured for 48 h. Luciferase activity was determined by a chemiluminescence analyzer. (**B**) RNA levels of Oct4 and Stat1 were detected by quantitative real-time RT-PCR in A549 and H1299 cells transduced with lentiviral vectors encoding Oct4. (**C**) Detection of Stat1 expression by immunoblotting in Oct4-overexpressing A549 and H1299 cells. Data are the mean ± S.E.M. ** *p* < 0.01 and *** *p* < 0.001.

To explore the potential relationship between Oct4 and Stat1, their promoter regions were analyzed. The results reveal that Oct4 may bind to the *Stat1* promoter. We examined whether Oct4 can transactivate the *Stat1* promoter using the luciferase reporter assay. The result show that Oct4 can enhance *Stat1* promoter activity in both A549 and H1299 cells (Figure 2A). Simultaneously, Oct4 upregulated the RNA level of Stat1 (Figure 2B). The protein level of Stat1 also increased in Oct4-overexpressing cells (Figure 2C).

**Figure 3:**
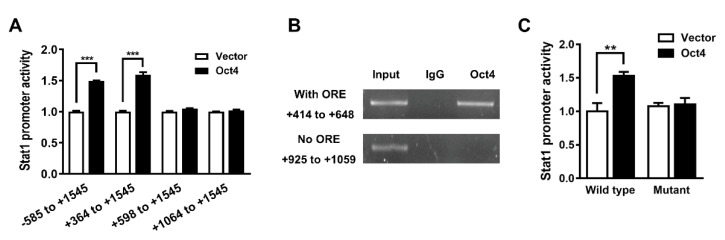
Oct4 directly binds to the *Stat1* promoter to modulate Stat1 expression. (**A**) A549 cells were transfected with pSin-EF2-Oct4-Pur and different deletion forms of the *Stat1* promoter, termed from -585 to +1545, from +364 to +1545, from +598 to +1545 and from +1064 to +1545, were transfected into A549 cells. Total cell lysates were collected at 48 h after transfection and their luciferase activities were determined by a chemiluminescence analyzer. (**B**) ChIP assay was performed in A549 cells using anti-Oct4 antibody. Normal IgG served as negative control. (**C**) The transactivation activity of wild-type and mutant *Stat1* promoters was determined in Oct4-overexpressing A549 cells. Data are the mean ± S.E.M. ** *p* < 0.01 and *** *p* < 0.001.

Next, we serially deleted four putative OREs on the *Stat1* promoter in order to determine the location of the ORE that is important for Oct4 to bind to and transactivate the *Stat1* promoter. Deletion of the promoter region between +364 and +598 bp significantly eliminated the responsiveness of the *Stat1* promoter to Oct4, suggesting that the response element within this region may be involved in Oct4-induced transactivation of the promoter (Figure 3A).

In addition, the ChIP assay revealed that Oct4 can bind to the Oct4 response element region (from +414 to +648 bp) but not to the non-response element region (from +925 to +1059 bp) within the *Stat1* promoter (Figure 3B). Furthermore, we used PROMO, a computer software, for predicting transcription-factor binding sites [30], to design a mutant sequence of the ORE (from ATTTGAAT to AGTTGAAT; from +576 to +583 bp). The point mutation of the potential response element within the *Stat1* promoter eliminated Oct4-mediated transactivation, indicating that this ORE is involved in Oct4-induced transactivation of the *Stat1* promoter (Figure 3C). Taken together, these results indicate that Oct4 can bind to the region between +576 and +583 bp in the *Stat1* promoter, increasing Stat1 expression.

### 3.3. Oct4 Modulates Anti-Apoptosis via the Stat1 Downstream Gene Mcl-1 and Inhibition of Stat1 Expression Induces Apoptosis

**Figure 4:**
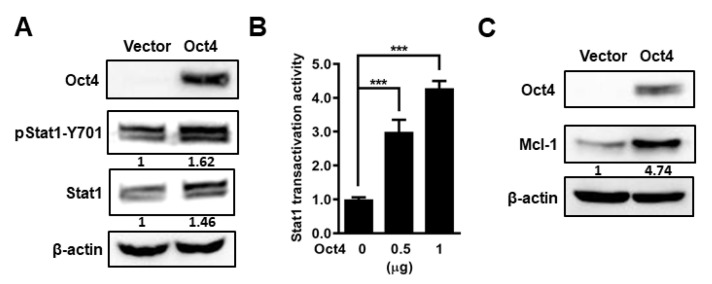
Oct4 increases Mcl-1 expression. (**A**) Detection of total and phospho-Stat1 by immunoblotting in Oct4-overexpressing A549 cells. (**B**) Stat1 transactivation activity was determined using luciferase assay in Oct4-overexpressing cells. (**C**) Mcl-1 expression was examined in Oct4-overexpressing A549 cells. Data are the mean ± S.E.M. *** *p* < 0.001.

As Stat1 phosphorylation is essential for regulating its downstream genes, we further explored whether Oct4 can enhance Stat1 phosphorylation. Oct4 overexpression increased not only Stat1 expression but also Stat1 phosphorylation on tyrosine 701 (Figure 4A). We next evaluated the transactivation of Stat1 using the Stat1 binding motif reporter construct pISRE-Luc. The luciferase reporter assay demonstrated that the transactivation activity of Stat1 significantly increased in Oct4-overexpressing cells, compared with control cells (Figure 4B). Mcl-1 is a survival factor and is regulated by Stat1 through its ISRE motif within the promoter region [31]. Mcl-1 expression was also upregulated in Oct4-overexpressing cells (Figure 4C).

**Figure 5:**
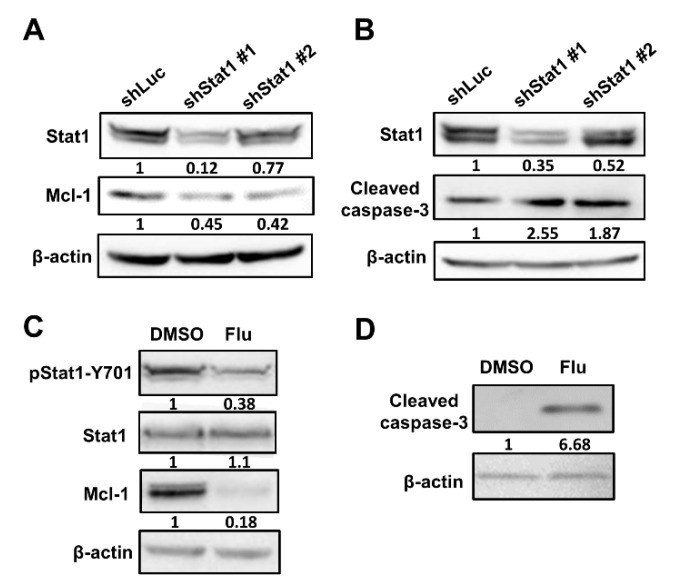
Inhibition of Stat1 sensitizes lung cancer cells to cisplatin-induced apoptosis. (**A**) Detection of Mcl-1 by immunoblotting in A549 cells transduced with lentiviral vectors expressing shStat1. (**B**) Stat1-knockdown cells were treated with cisplatin (5 μg/mL) for 24 h. Cleavage of caspase-3 was examined by immunoblotting. (**C**) A549 cells were treated with the Stat1 inhibitor fludarabine (Flu, 2.5 μg/mL) for 24 h. Detection of Mcl-1, as well as total and phospho-Stat1, by immunoblotting. (**D**) Cleavage of caspase-3 was detected in Flu-treated A549 cells.

Cisplatin can induce cell apoptosis and has been used clinically for nearly 30 years as part of the treatment package in many cancers. The use of cisplatin in chemotherapy is limited by the acquired or intrinsic resistance of cancer cells to the drug. As Mcl-1 plays a pivotal role in protecting cells from apoptosis, we explored the anti-apoptotic effect of Stat1. We confirmed that Mcl-1 expression reduced in Stat1-knockdown A549 cells (Figure 5A). Stat1 knockdown increased the cleavage of caspase-3 in cancer cells treated with cisplatin for 24 h (Figure 5B). To further confirm the anti-apoptotic role of Stat1, the Stat1 inhibitor fludarabine was used. After treatment with fludarabine, Stat1 phosphorylation and Mcl-1 expression decreased (Figure 5C), which induced cleavage of caspase-3 (Figure 5D). Taken together, the knockdown of Stat1 expression enhances cell apoptosis.

### 3.4. Silencing of Stat1 Expression Ameliorates Oct4-Induced Anti-Apoptosis

**Figure 6:**
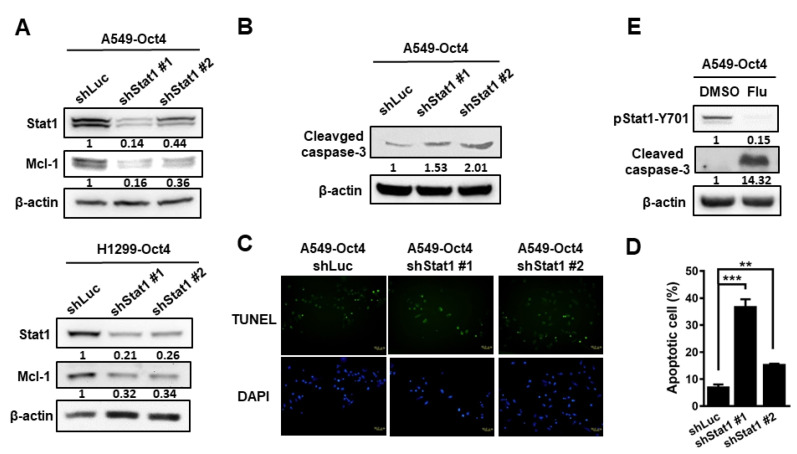
Oct4 suppresses apoptosis through Stat1. (**A**) Oct4-overexpressing A549 and H1299 cells were transduced with lentiviral vectors expressing shStat1. Mcl-1 expression was examined by immunoblotting in the transduced cells. (**B**) After treatment with cisplatin (5 μg/mL), cleavage of caspase-3 was detected in the transduced cells. (**C**,**D**) Apoptotic cells were stained using TUNEL assay (C) and counted (D) in the transduced cells treated with cisplatin. Scale bar = 50 μm; original magnification, ×200. **(E)** Oct4-overexpressing A549 cells were treated with fludarabine (Flu, 2.5 μg/mL) for 24 h and cleaved caspase-3 was detected by immunoblotting. Data are the mean ± S.E.M. ** p < 0.01 and *** p < 0.001.

As both Oct4 and Stat1 decreased cell apoptosis, we further investigated whether Oct4-regulated Mcl-1 expression is mediated by Stat1. Mcl-1 expression decreased in Oct4-overexpressing A549 and H1299 cells after Stat1 knockdown (Figure 6A). Cleavage of caspase-3 also increased after treatment with cisplatin to induce apoptosis in Stat1-knockdown cells, compared with control cells (Figure 6B). More apoptotic cells were detected in Stat1-knockdown cells than in control cells after treatment with cisplatin, as determined by the TUNEL assay (Figure 6C,D). Similarly, more cleaved caspase-3 was detected in fludarabine-treated cells than in control cells (Figure 6E). Taken together, Oct4 transactivates Stat1 expression and consequently enhances Mcl-1 expression, thus preventing cisplatin-induced apoptosis. Furthermore, suppression of Stat1 expression can sensitize lung cancer cells to cisplatin-induced cell death.

### 3.5. Oct4-Promoted Tumor Growth Is Attenuated by The Reduction in Stat1 Expression in Mice

**Figure 7:**
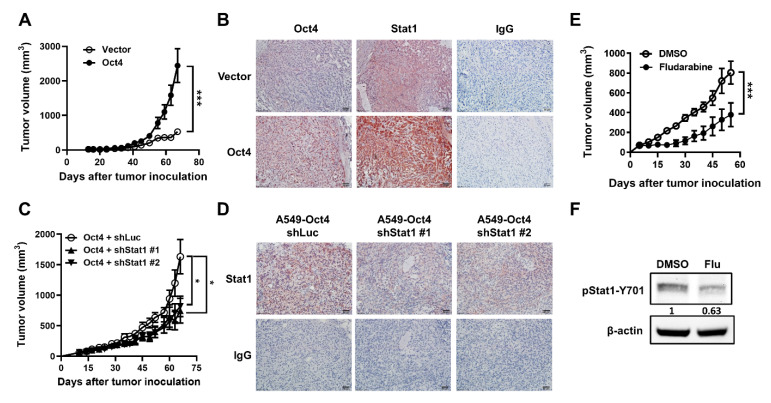
Oct4-promoted tumor growth is attenuated by reducing Stat1 expression. (**A**) NOD/SCID mice were subcutaneously inoculated with A549-Oct4 or A549-vector cells (*n* = 9). The tumor volume was measured every 3 days. (**B**) Detection of Oct4 and Stat1 by immunohistochemical staining in tumor tissues. Scale bar = 50 μm; original magnification, ×200. (**C**) Tumor-bearing mice were intratumorally treated with 1 × 10^9^ LPs of lentiviral vectors expressing shRNA specific to Stat1 at day 21 (*n* = 9 for each group). Tumor growth was monitored three times a week. (**D**) Detection of Oct4 and Stat1 by immunohistochemical staining in tumor tissues. Scale bar = 50 μm; original magnification, ×200. (**E**) Tumor-bearing mice were intratumorally treated with fludarabine (Flu) at day 14 (*n* = 8 for each group). (**F**) Detection of phospho-Stat1 by immunoblotting in tumor tissues. Data are the mean ± S.E.M. * *p* < 0.05 and *** *p* < 0.001.

To further demonstrate that Oct4 regulates Stat1 expression in vivo, tumor growth was monitored in mice subcutaneously inoculated with Oct4-overexpressing A549 cells. The tumor volume significantly increased in mice bearing Oct4-overexpressing tumors, compared with those bearing tumors transduced with the control vector (Figure 7A). In the tumor section excised at day 70 after tumor cell inoculation, Stat1 expression was higher in A549-Oct4 tumors than in A549-vector tumors, suggesting that Oct4 regulates Stat1 expression in vivo (Figure 7B). To clarify whether Oct4 promotes tumor growth through Stat1 in the mouse model, intratumoral injections of LV-shStat1 or LV-shLuc were administered in mice at 21 days following subcutaneous inoculation with Oct4-overexpressing A549 cells. The tumor volume significantly decreased in the Stat1-knockdown group, compared with the control group (Figure 7C). Immunohistochemical staining of tumor sections clearly revealed that lentiviral delivery of shRNA specific to Stat1 is effective in suppressing Stat1 expression (Figure 7D). Accordingly, treatment with the Stat1 inhibitor fludarabine at day 14 also inhibited tumor growth (Figure 7E) and reduced Stat1 phosphorylation (Figure 7F). Taken together, Oct4 overexpression results in a higher tumor growth rate, which can be attenuated by Stat1 inhibition.

## 4. Discussion

Previous studies have shown that Oct4 and Stat1 are overexpressed in lung cancer; however, their roles and underlying mechanisms are unclear [7,16]. In this study, we show that Oct4 expression is associated with Stat1 expression. In vitro studies indicate that Oct4 induces Stat1 expression by directly binding to the *Stat1* promoter, resulting in increased expression of Stat1 and the downstream gene Mcl-1. Furthermore, silencing of Stat1 expression inhibits tumor cell growth and sensitizes cancer cells to drug-induced apoptosis. Our results suggest that Oct4 acts in apoptosis through the regulation of Stat1 in lung adenocarcinoma.

Stat1 functions as a mediator of interferon signaling and is thought to be an inducer of cell death [13]. Stat1 overexpression has also been reported in many cancer types [15,16,17]. High Stat1 expression exhibits resistance to genotoxic stress following treatment with doxorubicin and cisplatin or a combination of ionizing radiation in cancer cells [18]. In this study, we show that Stat1 plays an anti-apoptotic role in lung adenocarcinoma. This might suggest that inhibition of apoptosis via inducing Mcl-1 expression protects cancer cells from stress in Stat1-overexpressing cells. Here, we propose a novel signaling pathway of Oct4 in cell survival, in addition to Stat3/survivin signaling in murine embryonic stem cells and chemoresistant colorectal cancer cells [32,33]. Oct4 directly binds to the *Stat1* promoter to induce Stat1 expression and further inhibits apoptosis. Activated Stat1 forms dimers with Stat3 to regulate gene expression [34]. Therefore, the exact involvement of Stat1 in Oct4/Stat3/survivin signaling remains to be elucidated.

In addition to anti-apoptosis, Oct4 is involved in cell migration and metastasis. Oct4 overexpression promotes migration in bladder cancer cells. Oct4 upregulates the expression of several genes, including fibroblast growth factor-4 (FGF-4), matrix metalloproteinase-2 (MMP-2), MMP-9 and MMP-13, which are known to contribute to metastasis [6]. Whether Stat1 participates in FGF-4 or MMP regulation needs to be further investigated. Moreover, previous studies have shown that Stat1 is involved in metastasis. Stat1 knockdown reduces the metastatic capacity of melanoma cells [15]. Furthermore, Stat1 directly interacts with focal adhesion kinase (FAK), which promotes cell motility [35]. Therefore, in addition to its anti-apoptotic properties, Stat1 might contribute to the regulation of focal adhesion and the promotion of tumor migration in lung adenocarcinoma. Whether Oct4 regulates FAK or is involved in focal adhesion requires further study.

Oct4 is involved in maintaining the self-renewing capacity and pluripotency of embryonic stem cells [3,4,5]. During embryonic development, microRNAs play an important role in regulating differentiation, especially miR-145. miR-145 promotes cell differentiation by targeting Oct4 on the 3′ untranslated region (UTR) [36,37]. Moreover, miR-145 expression is downregulated and is associated with poor differentiation and prognosis in non-small-cell lung cancer [38]. This provides a possible regulatory mechanism for Oct4 in lung cancer. Furthermore, miR-145 is regulated by interferon in a Stat1-dependent manner. miR-145 expression increases in Stat1-knockdown and Stat1-inhibitor-treated cells [39]. In our study, we found that Oct4 induces Stat1 expression and phosphorylation. Therefore, activated Stat1 might suppress miR-145 expression and further increase Oct4 expression to form a feedback loop in promoting tumor progression.

Fludarabine was popular in the past two decades for the treatment of chronic lymphocytic leukemia, before newer therapies were discovered. However, the results of clinical trials were generally disappointing, in particular phase II trials on major tumor types. Only in the cases of head and neck and breast cancers did a small proportion of patients show objective remissions [40,41]. A statistical framework based on the meta-analysis of expression profiles to identify pan-cancer markers and mechanisms of drug response using large panels of cancer cell lines from numerous distinct lineages characterized that the constitutive activation of several signaling pathways, including the interferon/Stat1 pathway, is implicated in resistance to the pan-histone deacetylase (HDAC) inhibitor [42]. Although phase III randomized studies do not support the use of HDAC inhibitors in lung cancer patients in routine practice, numerous preclinical studies have shown that HDAC inhibitors exhibit impressive antitumor activity in lung cancer cell lines [43]. In addition, cancer cells initially characterized as sensitive to chemotherapy may acquire resistance to chemotherapy and lead to chemotherapeutics-induced Oct4 expression, which contributes to drug resistance and tumor recurrence [44]. In this study, we found that Oct4 regulates Stat1 expression, which leads to cell survival via enhanced Mcl-1 expression and promotes tumor growth in a Stat1-dependent manner. In addition to being a DNA synthesis inhibitor, fludarabine, acting as a selective Stat1 activation inhibitor [45], may be a potential therapeutic agent in combination therapy for lung adenocarcinoma by inhibiting the anti-apoptotic effects of Oct4.

## 5. Conclusions

Tumor cells usually proliferate indefinitely through their anti-apoptotic properties. Evasion of cell death leads to ineffective therapy and poor prognosis in cancer patients. In our study, we demonstrated that the stem cell marker Oct4 is overexpressed and, ultimately, results in an anti-apoptosis phenotype through the Stat1/Mcl-1 axis in lung adenocarcinoma. Oct4 promotes tumor growth, whereas silencing of Stat1 expression reduces tumor growth in vivo. Moreover, Stat1 knockdown in Oct4-overexpressing cells sensitizes them to cisplatin-induced apoptosis. These results elucidate the molecular mechanism underlying Oct4-mediated cell survival in lung adenocarcinoma and provides broad implications for the concept and potential therapeutic applications.

## Data Availability

All data generated or analyzed during this study are included in this published article.
